# Maternal Vitamin D Status at Week 30 of Gestation and Offspring Cardio-Metabolic Health at 20 Years: A Prospective Cohort Study over Two Decades

**DOI:** 10.1371/journal.pone.0164758

**Published:** 2016-10-20

**Authors:** Dorte Rytter, Bodil Hammer Bech, Thorhallur Ingi Halldorsson, Tine Brink Henriksen, Charlotta Grandström, Arieh Cohen, Sjurdur Frodi Olsen

**Affiliations:** 1 Department of Public Health, Section for Epidemiology, Aarhus University, Aarhus, Denmark; 2 The Unit for Nutrition Research, Faculty of Food Science and Nutrition, School of Health Sciences, University of Iceland, Reykjavik, Iceland; 3 Centre for Fetal Programming, Statens Serum Institut, Copenhagen, Denmark; 4 Pediatric Department, Aarhus University Hospital, Aarhus, Denmark; 5 Clinical Mass Spectrometry Department of Clinical Biochemistry, Immunology and Genetics, Statens Serum Institut, Copenhagen, Denmark; University of Alabama at Birmingham, UNITED STATES

## Abstract

**Background/Objectives:**

Vitamin D deficiency is common among pregnant women and since the fetus relies exclusively on maternal supply, deficiency could potentially interfere with fetal development. Vitamin D blood concentrations during pregnancy have been associated with offspring cardio-metabolic health in a few previous studies but the evidence is still inconsistent and only one previous study has followed the offspring into adulthood. The aim of the present study was to investigate the association between maternal serum concentration of vitamin D (25(OH)D) in week 30 of gestation and offspring cardio-metabolic risk factors at 20 years.

**Subjects/Methods:**

A follow up study of a Danish birth cohort from 1988–89 (n = 965) was conducted. A blood sample was drawn from the women in week 30 of gestation. In 2008–2009, 95% of the original mother and child dyads could be identified in the central registration registry and were alive and living in Denmark. The offspring were followed up with self-reported anthropometrics (N = 629, 69%) and a clinical examination (N = 410, 45%). Multiple linear regression was used to estimate the association between maternal 25(OH)D and offspring cardio-metabolic risk factors adjusting for potential confounders.

**Results:**

No overall association was observed between maternal 25(OH)D in week 30 of gestation and offspring cardio-metabolic risk factors. However, the analyses did suggest a possible inverse association with blood pressure in females.

**Conclusions:**

No clear association between maternal 25(OH)D concentration in week 30 of gestation and cardio-metabolic risk factors in the 20 year old offspring was found.

## Introduction

Sub-optimal vitamin D status is common among pregnant women [1–4] and since the fetus relies exclusively on maternal supply of vitamin D across the placenta [5], maternal vitamin D deficiency could potentially interfere with fetal development.

Vitamin D is a steroid hormone which regulate a large number of genes through the binding to vitamin D receptors (VDR) [6]. These receptors are present in most human tissues and have been shown to be involved in a number of physiological processes [6]. The widespread distribution of VDRs and multiple actions of vitamin D makes it plausible that vitamin D insufficiency during fetal life could lead to perturbations in physiological processes and organ development which ultimately may lead to future disease. Accordingly, low vitamin D status during fetal life has been suggested to be associated with a number of adverse long term outcomes including asthma and allergies, bone mineralization, type 1 diabetes, schizophrenia, multiple sclerosis and cardio-metabolic health[7–24].

The association between vitamin D status in early life and later cardio-metabolic risk factors in the offspring has only been investigated in few studies and findings are still inconclusive. Hence, whereas some studies report increased adiposity in individuals exposed to low vitamin D concentrations during fetal or neonatal life [20], others report the opposite [21] or no association [22, 23]. Findings seem to vary inconsistently with sex. Similar inconsistent findings have been reported for other cardio-metabolic risk factors such as fasting insulin, triglycerides, cholesterol, CRP and blood pressure [20–24]. Most studies only follow-up individuals into early childhood and only one study has previously followed the participants into adulthood [21].

The objective of this study was to investigate the association between maternal 25(OH)D concentration in week 30 of gestation and offspring cardio-metabolic health at 20 years of age in a prospective cohort study.

## Materials and Methods

### Original study

Details about the recruitment have been described in detail previously [25]. Briefly, the original study population included 965 pregnant women recruited for a birth cohort study in Aarhus, Denmark from April 1988 until January 1989. These women represented 80% of a consecutive sample of 1212 women attending routine antenatal care at a midwife center in the city. In week 30 of gestation, the women filled out a dietary questionnaire also containing information about lifestyle and education and an accompanying interview was undertaken. A venous blood sample was obtained after the interview. The blood sample was processed and serum was frozen at -20°C. The mean age of the participating women was 29 (SD: 4) years, approximately 58% were nulliparous and 40% had been smoking during pregnancy.

### The follow up

The follow-up of the offspring has been described in detail previously [26]. In short, offspring were followed up in 2008–2009 both with a web-based questionnaire and a clinical examination. In addition to questions regarding health and lifestyle, the questionnaire included questions about height, weight and waist circumference. The clinical examination included measurement of anthropometry, blood pressure and heart rate. Additionally, a fasting venous blood sample was drawn. Blood sample analyses included measurement of glucose, insulin, HbA1c, Insulin-like Growth Factor (IgF1), leptin, adiponectin, cholesterol, triglycerides and high-sensitivity C-reactive Protein (hs-CRP). Insulin resistance was estimated by HOMA_IR (fasting insulin (μU/mL) x fasting glucose (mmol/L)/22.5). Details about the physical examination and biochemical analyses have been published previously [26].

### The study sample

A total of 915 (95% of the original cohort) mother and child dyads could be identified in the central registration registry and were alive and living in Denmark at the time of the follow-up. A sufficient amount of serum for the vitamin D (25(OH)D) analysis was available in 851 mothers of which one outlying observation ([25(OH)D] = 366nmol/l) was excluded from analysis. Of the 850 offspring available for follow-up, a total of 629 (74%) gave information about self-reported anthropometrics and 410 (48%) participated in the clinical examination. A flow chart for the study population is included as **S1 Fig**.

### Exposure assessment

Maternal concentrations of 25(OH)D were quantified in 2010 from the serum samples drawn in gestational week 30 using the LC-MS/MS method (‘MS/MS vitamin D’ kit from Perkin Elmer, Waltham, MA) [27]. The method of analysis has been described previously [28]. 25(OH)D has been shown to be very stable when stored in the freezer[29].

Of the 649 mothers with measured serum 25(OH)D who had children participating in the follow-up, 10 mothers had a 25(OH)D_3_ concentration below the level of quantification (LOQ<8.5nmol/L). A concentration of 4.25 nmol/L was imputed for these subjects. Also, 529 mothers had a 25(OH)D_2_ concentration below the LOQ (5.9nmol/L). Since 25(OH)D_2_ concentrations generally were low compared to 25(OH)D_3_, a 25(OH)D_2_ concentration of 0 nmol/L was imputed for these subjects. This very high prevalence of low 25(OH)D_2_ concentration was expected since Danish women do not have a tradition of taking supplements containing 25(OH)D_2_.

Total serum 25(OH)D concentration, calculated as the sum of 25(OH)D_2_ and 25(OH)D_3_, was used as a continuous measure of exposure in the analyses. Differences in outcomes are reported per 50 nmol/L increase in maternal total 25(OH)D concentration. As most women had a 25(OH)D_2_ concentration below LOQ, imputing 0 and adding this to the concentration of 25(OH)D_3_ may lead to biased exposure estimates. In order to examine the stability of this approach additional analyses were run where only the concentration of 25(OH)D_3_ was used as exposure.

### Assessment of cardio-metabolic risk factors

Since information on self-reported height, weight and waist circumference was available in more subjects compared to the clinically measured information, the former was used in the analyses concerning BMI and waist circumference (n = 623 and 629 respectively). Blood pressure, heart rate and all biochemical measures were only available for those participating in the clinical examination (n = 410).

### Covariates

Information on age and parity as well as maternal pre-pregnancy height and weight was collected from hospital records. Maternal prepregnancy height and weight was used to calculate maternal prepregnancy BMI. Information on maternal smoking and educational level during pregnancy was obtained from the dietary questionnaire and interview in week 30 of gestation. In the Danish Medical Birth Registry we obtained information about offspring birth weight and gestational age.

Information on offspring exercise habits and lifestyle was obtained from the web-based questionnaire at follow-up.

The study was conducted according to the guidelines given in the declaration of Helsinki and all procedures involving human subjects were approved by the Central Denmark Region Ethics committee (journal no. 20070157) and the Danish Data Protection Agency (journal no. 2006-41-6257). Written informed consent was obtained from all participants.

### Statistical analyses

Multiple linear regression modeling was used to estimate the association between maternal serum 25(OH)D and the different cardio-metabolic risk factors, treating 25(OH)D as a continuous variable.

Sixteen percent of all participants had an hs-CRP value below the detection limit of 0.20 mg/L. Assuming the data below the detection limit were missing at random, multiple imputations were used to impute the values below the detection limit. A maximum value of 0.2 mg/L was set and the variables sex, BMI, smoking habit and parental overweight were thought to provide sufficient information on hsCRP to impute the missing values. 20 rounds of multiple imputation were run, using the Stata command ice (imputation by chained equations).

The distribution of most biochemical outcomes was skewed and therefore log transformed in order to normalize the distributions. Following this, all regressions were found to fulfill the assumptions of normally distributed residuals and approximately equal variance of residuals.

It was a priori decided to adjust for major risk factors for the outcome. Hence, maternal pre-pregnancy BMI (4–5% missing), maternal education (elementary school, high-school or technical school, university education, higher academic education or other education, 7–9% missing) and smoking during pregnancy (never, <10 and ≥10 cigarettes/day, 4–5% missing) was included in the model to adjust for potential genetic and/or environmental disposition to the outcomes. Also, maternal age (1% missing), and parity (1.0% missing) could be associated with vitamin D concentration and was included to adjust for factors that could indirectly affect adiposity. Potential risk factors such as offspring exercise habits and lifestyle was not included in the model, since information about these covariates was collected cross-sectionally. A Directed Acyclic Graph displaying the potential confounders is presented in S2 Fig.

Differential programming effects have previously been described in males and females and therefore all analyses were initially stratified by sex [30]. Sex specific associations are reported for anthropometric measures, blood pressure and heart rate. However, since the estimated associations in males and females were not statistically significantly different for most of the biochemical outcomes, sex was included as a covariate in these models and results are presented for both sexes combined for all biochemical outcomes except HDL cholesterol. Only participants with complete information on covariates were included in the main analyses.

We did not adjust for season of maternal blood collection. However, since season may be associated with lifestyle, diet and exercise levels atweek 30 of gestation, which could potentially affect the outcomes, sub-analyses were done including season as a covariate.

Due to the relatively high loss to follow-up and due to a possible association between participation and offspring BMI, sensitivity analyses were performed for BMI and blood pressure. This was done by using multiple imputation. Hence, assuming the values missing, were missing at random, multiple imputation was used to impute the values based on sex, maternal pre-pregnancy BMI, parity, maternal smoking during pregnancy, maternal education and offspring birth weight. In addition, the imputation of blood pressure was also based on self-reported height and BMI. Since information used to impute the values was sparse an additional sensitivity analysis for BMI was performed where a BMI of 25 kg/m^2^ was imputed for those with missing outcome information.

All statistical tests were performed using STATA software package 13.0 (Stata corporation, College Station, Texas).

All tests were two-sided and a *P*-value below 0.05 was considered statistically significant.

## Results

Mothers excluded from analyses due to missing exposure information were similar to participating mothers with regard to most covariates **(S1 Table).** However, birth weight was lower in offspring of mothers excluded (3.33 (SD: 0.60) kg vs. 3.51 (SD: 0.52) kg). Participants in the follow-up (clinical and/or questionnaire) differed from non-participants with regard to a number of covariates. Hence, non-participants were more likely to have a lower birth weight, be born of mothers who were less educated, had a higher pre-pregnancy BMI and who were smoking during pregnancy. Also, participants only filling out the questionnaire were more likely to be male, had a higher self-reported BMI and exercised less compared to participants in the clinical examination. Participants and non-participants did not differ with regard to maternal 25(OH)D concentration. Approximately 25% of participants in the follow-up were exposed to maternal 25(OH)D concentrations below 50 nmol/L.

A description of the associations between maternal and offspring covariates with maternal 25(OH)D is shown in **Table 1**. Parity and pre-pregnancy BMI as well as prevalence of parental overweight reported by the offspring at follow-up, tended to be higher in mothers in the lowest quartile of 25(OH)D. Prevalence of maternal smoking tended to increase from higher to lower quartiles of 25(OH)D, whereas birth weight tended to increase with an increase in 25(OH)D.

**Table 1:** Maternal and 20 year old offspring characteristics dependent on quartiles of maternal 25(OH)D concentration at week 30 of gestation^1^

		1^st^ quartile	2^nd^ quartile	3^rd^ quartile	4th quartile
**Maternal characteristics**^2^					
Vitamin D, nmol/L		33 ± 13	63 ± 8	92 ± 9	133 ± 26
Maternal smoking, yes		63 (43)	57 (37)	50 (34)	55 (34)
Parity					
0		70 (46)	92 (58)	103 (66)	108 (64)
1		66 (44)	51 (32)	41 (26)	44 (26)
≥2		15 (10)	17 (11)	12 (8)	16 (10)
Pre-pregnancy BMI, kg/m^2^		21.8 ± 3.8	21.1 ± 2.8	21.4 ± 2.5	20.9 ± 2.2
Age, years		29.0 ± 3.8	29.4 ± 4.1	29.2 ± 4.2	29.1 ± 3.8
Maternal education					
	Elementary school	21 (15)	12 (8)	11 (8)	17 (11)
	High-school or technical school	24 (17)	37 (25)	35 (24)	41 (26)
	University	57 (41)	56 (37)	58 (40)	64 (41)
	Higher academic	24 (17)	37 (25)	30 (21)	22 (14)
	Other	12 (9)	9 (6)	11 (8)	11 (7)
**Offspring characteristics**^3^					
Birth weight, kg		3.50 ± 0.57	3.56 ± 0.47	3.52 ± 0.51	3.58 ± 0.50
Gestational age, days		283 ± 11	282 ± 13	283 ± 11	284 ± 11
Sex, male		72 (46)	76 (47)	78 (50)	80 (47)
Offspring smoking					
	Current	31 (20)	25 (16)	31 (20)	30 (18)
	Ex-smoker	7 (5)	7 (5)	5 (3)	7 (4)
	Occasional	38 (25)	30 (19)	45 (29)	40 (24)
	Never	79 (51)	92 (60)	72 (47)	89 (54)
Parental over-weight^4^		65 (45)	49 (32)	59 (40)	54 (34)
Self-reported BMI, kg/m^2^		22.2 ± 2.8	21.8 ± 2.6	22.4 ± 3.3	21.9 ± 2.8
Exercise, yes		108 (69)	113 (72)	110 (71)	126 (76)
Strenuous exercise^5^		88 (59)	94 (61)	94 (63)	99 (63)

^1^ Data are means ± SD or n (%).

^2^ Information collected from a self-administered questionnaire and structured interview of the pregnant women in week 30 of gestation.

^3^ Information collected from a self-administered web-based questionnaire to the offspring at the age of 19–20 y. Gender, birth weight and gestational age collected from birth records.

^4^ Participants were asked whether they would consider any of their parents to be overweight.

^5^ Defined as exercise of at least 20 minutes duration, resulting in breathlessness.

**Fig 1:**
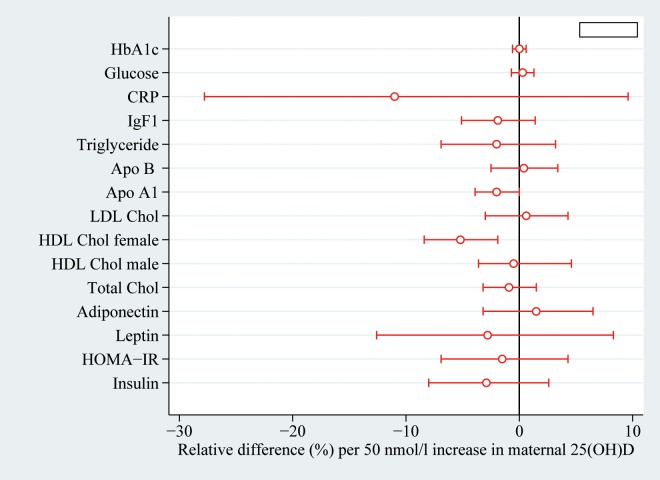
Relative difference in offspring cardio-metabolic risk factors measured at 20 years per 50nmol/l increase in maternal 25(OH)D in week 30 of gestation (n = 368). Estimates are adjusted for maternal age, parity, pre-pregnancy BMI, smoking during pregnancy and offspring sex. Horizontal lines indicate 95% confidence intervals. Abbreviations: HbA1c: Glycated hemoglobin, CRP: C-reactive protein, IgF-1: Insulin-like growth factor-1, HOMA-IR: Homeostatic model assessment of insulin resistance.

Maternal 25(OH)D concentration was not associated with BMI, waist circumference or the adipose tissue derived hormones leptin and adiponectin in the 20 year old offspring independent of sex (**Table 2, Fig 1**). Also, no association was found with glucose metabolism measured by fasting glucose, insulin, HOMA-IR and HbA1c (Fig 1). Regarding lipid metabolism, no association was found with total and LDL cholesterol, triglyceride and apolipoprotein B (Fig 1). However, a week inverse association between 25(OH)D and HDL cholesterol was found in females (Fig 1). Offspring IgF-1 was not associated with maternal 25(OH)D during pregnancy. Also, although the estimated difference in offspring hsCRP per 50 nmol increase in maternal 25(OH)D was relatively large, the confidence interval was wide and the association was found statistically insignificant (Fig 1).

**Table 2:** Sex-specific association between maternal 25(OH)D concentration in week 30 of gestation and offspring adiposity, blood pressure and heart rate at age 20y^1^.

	Difference per 50 nmol/l increase in 25(OH)D concentration	
	Crude	Adjusted^2^
**Males**		
BMI (kg/m^2^)^3^	-0.1 (-0.5;0.3)	0.0 (-0.4; 0.5)
Waist circumference (cm)^4^	-0.6 (-2.0; 0.8)	-0.1 (-1.5; 1.3)
Systolic BP (mmHg)^5^	-0.7 (-2.4; 1.0)	-0.1 (-1.9; 1.7)^6^
Diastolic BP(mmHg)^5^	-0.4 (-1.7; 0.9)	-0.2 (-1.5; 1.2)^6^
Heart rate (beats per min)^5^	2.0 (0.2; 3.7)	2.6 (0.7; 4.4)*
**Females**		
BMI (kg/m^2^)^7^	-0.1 (-0.5; 0.3)	0.1 (-0.4; 0.5)
Waist circumference (cm)^8^	-0.6 (-1.9; 0.8)	0.1 (-1.3; 1.5)
Systolic BP (mmHg)^9^	-1.8 (-3.0; -0.5)*	-1.2 (-2.6; 0.1)^6^
Diastolic BP (mmHg)^9^	-1.20 (-2.2; -0.20)*	-0.9 (-2.0; 0.2)^6^
Heart rate (beats per min)^9^	-0.8 (-2.4; 0.7)	-0.6 (-2.3; 1.0)

* P<0.05

^1^ All values are differences in outcome measure per 50nmol/L difference in maternal 25(OH)D concentration with 95% confidence intervals in parentheses.

^2^ Adjusted for maternal pre-pregnancy BMI, age, parity, education and smoking as well as offspring sex.

^3^ n = 291 in crude analysis and 263 in adjusted analysis.

^4^ n = 297 in crude analysis and 268 in adjusted analysis.

^5^ n = 162 in crude analysis and 150 in adjusted analysis.

^6^Additionally adjusted for offspring height.

^7^n = 332 in crude analysis and 290 in adjusted analysis.

^8^ n = 332 in crude analysis and 292 in adjusted analysis.

^9^ n = 248 in crude analysis and 218 in adjusted analysis.

Further adjustment for season of blood sample collection tended to attenuate most of the associations (data not shown).

No association between 25(OH)D and blood pressure was found in males (Table 2). There was, however, a tendency towards an inverse association with systolic and diastolic blood pressure in females. Adjustment for season of collection of blood sample in the sex-stratified analyses further strengthened the association between maternal 25(OH)D and offspring systolic and diastolic blood pressure in females (systolic blood pressure -1.5 mmHg/50nmol/L (95% CI: -3.1, 0.1) and diastolic blood pressure -1.4 mmHg/ 50nmol/L (95% CI: -2.7, -0.0). With regard to heart rate, a statistically significant association was found in males but not in females.

Sensitivity analysis based on multiple imputation did not change the results for the association between maternal 25(OH)D concentration and offspring BMI and only slightly strengthened the association with blood pressure (data not shown). Using only 25(OH)D_3_ in the analyses did not change the results.

## Discussion

The results from this study indicate no clear association between maternal 25(OH)D concentration and offspring cardio-metabolic health. Hence, no association was found with offspring adiposity, adiposity derived hormones and glucose metabolism. Also, no statistically significant association was found with systolic and diastolic blood pressure, although the data suggested an inverse association in females. An indication of an increase in resting heart rate with increasing maternal 25(OH)D was however found in males. There was no consistent evidence for an association with total cholesterol, LDL cholesterol, Apolipoprotein B and IgF-1. However, maternal 25(OH)D concentration approached an inverse association with hsCRP and was inversely associated with offspring HDL in females.

Strengths of the study include the long term follow-up, the comprehensive outcome assessment and the relatively large cohort. However, the long term follow-up comes at a cost. Hence, the loss to follow-up was relatively large (26% for self-reported anthropometrics and 52% for the clinically measured outcomes). It is likely that loss to follow up was associated with BMI and therefore also a number of other cardio-metabolic risk factors. However, since loss to follow-up was not associated with 25(OH)D, the degree of selection bias most likely was limited. This is supported by the sensitivity analyses which did not change estimates materially.

Since the analyses were based on self-reports, we cannot dismiss the risk of information bias. The difference between self-reported and clinically measured BMI did not depend on maternal 25(OH)D (n = 389) indicating that differential misclassification was not responsible for the lack of association. However, the low reliability or precision of self-reported BMI and waist circumference could potentially have masked a true association with maternal 25(OH)D, although the very low estimated differences indicate that this was not the case.

In this study we only measured 25(OH)D as a precursor of the biologically active 1.25(OH)_2_D3. Other biologically active hydroxy-derivatives have been identified and the concentration of 25(OH)D may not be a sufficient measure of vitamin D deficiency [31–33]. However, the concentrations of these derivatives have been shown to be 15–30 fold lower than the concentration of 25(OH)D and the physiological importance still needs to be established.

Previous evidence on the association between maternal 25(OH)D concentration during pregnancy and offspring adiposity is inconsistent, with both inverse, direct and no association reported [20–23]. In a cohort study nested in the Southampton Women’s survey, lower maternal vitamin D status was associated with a greater fat mass at the age of 6 years [20]. Likewise, a study conducted in India found that intrauterine exposure to vitamin D deficiency (25(OH)D below 50 nmol/L) was associated with a higher fat mass and lower fat free mass in boys but not girls at the age of 5 years [22]. This association, however, did not persist at 9.5 years of age. In agreement with findings from the present study, a small UK cohort study found no statistically significant association between maternal vitamin D status and offspring anthropometry at 9 years [23]. However, the small number of participants examined at 9 years (n = 178) made it difficult to draw firm conclusions. Surprisingly, a study by Tornhammar et al found a direct association between neonatal 25(OH)D_3_ status and BMI at 35 years [21], but only approximately 26% of the invited chose to participate in the follow-up making the results very vulnerable to selection bias. BMI may be a rather poor measure of adiposity, especially in the physically active males. Hence, differences in the assessment of adiposity in the different studies could partly explain the different findings.

In the present study we found no statistically significant association between maternal 25(OH)D at week 30 of gestation and both diastolic and systolic blood pressure, however the estimates indicated an inverse association in females. Also, an indication of an association between maternal 25(OH)D and heart rate was found in males. Sex specific associations with maternal 25(OH)D have previously been described with respect to percentage body fat and HDL cholesterol [22]. The clinical relevance of the associations are likely marginal but given that blood pressure tracks into older ages, we cannot rule out that the difference increases with age.

Vitamin D deficiency has been shown to down-regulate the renin-angiotensin system [19]. The local renin-angitensin system (RAS) has been shown to be functional in the fetal brain in late gestation and to be important for the central regulation of blood pressure [34]. Also, RAS has been shown to be important in nephrogenesis [35]. Hence, through its effects on RAS, vitamin D deficeniency could have the potential to influence long term blood pressure control. However, the optimal timing of exposure assesment can be discussed. The concentration of vitamin D is highly dependent on season and a low concentration of vitamin D in week 30 of gestation could indicate a higher concentration in early gestation or vice versa.

The results from the present study on blood pressure are partly in agreement with results from a follow up of offspring from the ALSPAC study at the age of 9.9 and 15.4 years [24]. This study found an inverse association between maternal 25(OH)D concentration during pregnancy and systolic blood pressure at 9.9 years of follow-up but this association had disappeared when the offspring were reexamined at 15.4 years of age. An indication for an association between maternal 25(OH)D deficiency (25(OH)D<50 nmol/l) and offspring systolic blood pressure at 9.5 years was also found in the study by Krishnaveni et al. However, the difference between children of deficient and non-deficient mothers failed to reach statistical significance [22]. In two additional studies, no statistically significant association was found between maternal or infant 25(OH)D and blood pressure at 9 and 35 years, respectively [21, 23].

In the present study, no association between maternal 25(OH)D during pregnancy and offspring biochemical outcomes associated with glucose and lipid metabolism was found, with the exception of an inverse association with HDL and apolipoprotein A1 in females. Most other studies report no association with offspring HDL [21, 24], with the exception of Krishnaveni et al who also find an inverse association but only in 9.5 year old boys [22]. The discrepancy between the sex specific effects and the fact that many comparisons were made in the study, could indicate a chance finding. Other studies have reported an association between maternal 25(OH)D and offspring glucose metabolism [21, 22]. However both inverse and direct associations have been reported and in agreement with the present study, Williams et al reported no association with insulin, insulin resistance and fasting glucose concentrations.

Williams et al also found an indication for an inverse association between maternal 25(OH)D and offspring CRP concentration[24]. Like in the present study, the findings were however statistically insignificant and larger studies are needed before firm conclusions can be drawn.

In conclusion, no association between maternal 25(OH)D concentration at week 30 of gestation and cardio-metabolic risk factors in the 20 year old offspring was found. There was however indications of a possible sex specific inverse association with offspring blood pressure (females only).

## Supporting Information

S1 Fig Flow chart. (DOCX)

S2 Fig Directed Acyclic Graph. (PDF)

S1 Table Maternal and offspring characteristics at different levels of participation^1^. ^1^ Data are means ± SD or n(%). ^2^ Information collected from a self-administered questionnaire and structured interview of the pregnant women in week 30 of gestation. ^3^ Information collected from a self-administered web-based questionnaire to the offspring at the age of 19–20 y. Gender, birth weight and gestational age collected from birth records. ^4^ Participants were asked whether they would consider any of their parents to be overweight. ^5^ Defined as exercise of at least 20 minutes duration, resulting in breathlessness.
(PDF)
