# Supplementary material for: Maternal Vitamin D Status at Week 30 of Gestation and Offspring Cardio-Metabolic Health at 20 Years: A Prospective Cohort Study over Two Decades
Source: PLoS One. 2016 Oct 20;11(10):e0164758. doi: 10.1371/journal.pone.0164758 (PMC5072548; doi:10.1371/journal.pone.0164758)
Supplement: S1 Fig — (DOCX) [file pone.0164758.s001.docx]

**Supplemental Figure 1.** Flow chart

623 with information on BMI

629 with information on waist circumference

410 offspring participated in clinical examination

641 offspring filled out questionnaire

849 mothers with information about vitamin D concentration

1 mother with outlying Vitamin D values

15 children dead or abroad

27 mothers dead or abroad

2 twins

64 mothers with no blood sample

850 mothers with blood samples

915 mothers contacted

959 children identified

1 child CPR missing

960 mothers identified

5 mothers with wrong CPR no.

965 mothers enrolled
