# Supplementary figures and images for: Maternal Vitamin D Status at Week 30 of Gestation and Offspring Cardio-Metabolic Health at 20 Years: A Prospective Cohort Study over Two Decades
Source: PLoS One. 2016 Oct 20;11(10):e0164758. doi: 10.1371/journal.pone.0164758 (PMC5072548; doi:10.1371/journal.pone.0164758)

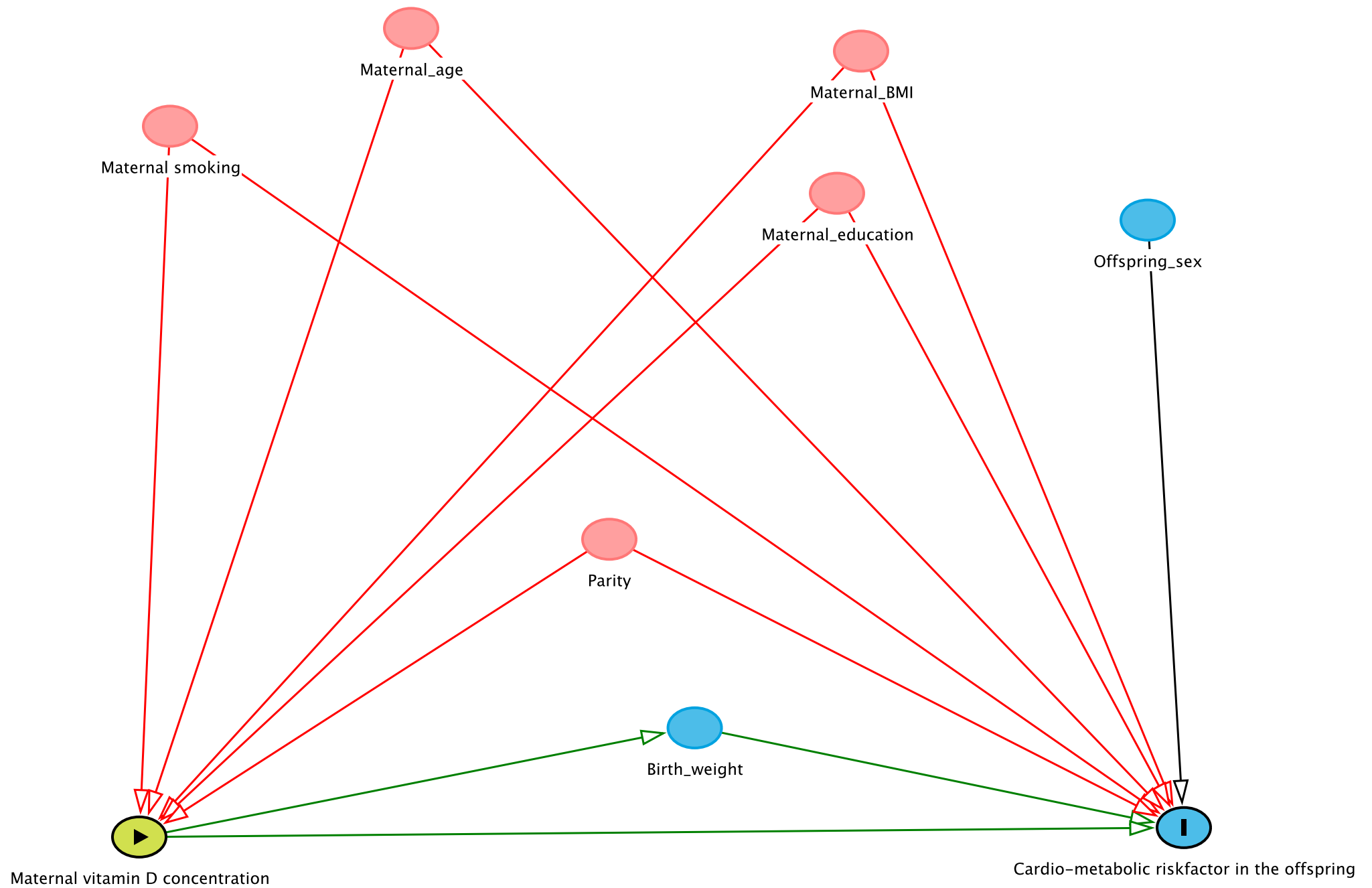

Supplement: S2 Fig — (PDF) [file pone.0164758.s002.pdf]
