# Supplementary material for: Maternal Vitamin D Status at Week 30 of Gestation and Offspring Cardio-Metabolic Health at 20 Years: A Prospective Cohort Study over Two Decades
Source: PLoS One. 2016 Oct 20;11(10):e0164758. doi: 10.1371/journal.pone.0164758 (PMC5072548; doi:10.1371/journal.pone.0164758)
Supplement: S1 Table — 1 Data are means ± SD or n(%). 2 Information collected from a self-administered questionnaire and structured interview of the pregnant women in week 30 of gestation. 3 Information collected from a self-administered web-based questionnaire to the offspring at the age of 19–20 y. Gender, birth weight and gestational age collected from birth records. 4 Participants were asked whether they would consider any of their parents to be overweight. 5 Defined as exercise of at least 20 minutes duration, resulting in breathlessness. (PDF) [file pone.0164758.s003.pdf]

	Level of participation			
Maternal/offspring characteristics	Clinical examination (410)	Questionnaire only (234)	No participation (206)	No exposure information (65)
**Maternal characteristics**[2]				
Smoking	146 (37%)	79 (36%)	98 (50%)	21 (39%)
Parity				
0	242 (60%)	131 (57%)	107 (54%)	40 (65%)
1	134 (31%)	78 (34%)	66 (34%)	15 (24%)
≥2	40 (10%)	20 (9%)	24 (12%)	7 (11%)
Prepregnancy BMI, kg/m^2	21.4 ± 3.1	21.1 ± 2.5	21.8 ± 3.8	21.6 ± (2.7)
Age, years	29.2 ± 4.0	29.1 ± 4.0	28.8 ± 4.6	29.0 ± 4.7
Education				
Elementary school	35 (9%)	26 (13%)	41 (22%)	7 (13%)
High-school or technical school	84 (22%)	53 (25%)	65 (35%)	12 (23%)
University	155 (41%)	80 (38%	54 (29%)	22 (42%)
Higher academic	75 (20%)	38 (18%)	11 (6%)	7 (13%)
Other	32 (8%)	11 (5%)	15 (8%)	5 (9%)
Vitamin D, nmol/L	82.6 ± 41.0	78.1 ± 39.2	78.6 ± 40.9	-
**Offspring**				

**characteristics**[3]				
Sex, male	162 (40%)	144 (62%)	139 (67%)	31 (48%)
Birth weight, g	3.5 ± 0.5	3.6 ± 0.5	3.4 ± 0.5	3.3 ± 0.6
Gestational age, days	283 ± 11.2	283 ± 12	282 ± 11	282.6 ± 12.5
Smoking				-
Current	69 (17%)	48 (21%)	-	-
Ex-	18 (5%)	8 (3%)	-	-
Occasional	104 (26%)	49 (21%)	-	-
Never	208 (52%)	124 (54%)	-	-
Parental overweight[4], yes	143 (37%)	84 (38%)	-	-
Self-reported BMI, kg/m^2	21.8 ± 2.8	22.5 ± 3.0	-	-
Exercise, yes	299 (75%)	158 (68%)	-	-
Strenous exercise[5], yes	248 (64%)	127 (57%)	-	-
